# EHMTI-0152. Epidemiology of primary headaches among adolescents in republic of Croatia

**DOI:** 10.1186/1129-2377-15-S1-B20

**Published:** 2014-09-18

**Authors:** M Sedlic, D Mahovic

**Affiliations:** 1 Neurology, Clinical Hospital Center Zagreb, Zagreb, Croatia

## 

The aim of our research was to perform an epidemiological study of migraine and tension headache among adolescents in Republic of Croatia and to determine whether there are differences in prevalence, socioeconomic status, headache onset, gender distribution, health care utilization and self-medication between these two types of primary headaches.

We have surveyed 1876 students attending 18 secondary schools in 10 cities of Croatia using self-administered questionnaire that was designed to classify respondents into a group of migraine or tension headache according to Lipton criteria for migraine and ICHD-2 criteria for both migraine and tension headache.

Prevalence of migraine was 12,8% (17% in women and 8,1% in men) and prevalence of tension headache was 38,3% (40,6% in women and 35,7% in men). Prevalence of migraine with TTH was 2,9% (3,1% in women and 2,7% in men). There was significant difference in prevalence of migraine (OR=2,3) and tension headache (OR=1,23) between female and male students. We have found that migraineurs were more prone to self-medication (OR=3,29), as to health care utilization (OR=8,12). Also we have identified smoking as a precipitating factor for migraine.

The prevalence of primary headaches in Croatia is similar to that in other countries of the world, tension headache is the most common primary headache, occurring later than migraine, and both types are more common in females. Smoking is a risk factor for developing migraine. Although migraineurs visit the doctor and take medications more frequently, both headaches are underdiagnosed and undertreated.

No conflict of interest.

